# Individualized connectomic tACS immediately improves oscillatory network with language facilitation in post-stroke aphasia: a feasibility study of a dysfunctome-based targeting approach

**DOI:** 10.3389/fncom.2025.1635497

**Published:** 2025-09-04

**Authors:** Chester Yee-Nok Cheung, Anthony Pak-Hin Kong, Mehdi Bakhtiar

**Affiliations:** ^1^The Aphasia Research and Therapy (ART) Laboratory, Academic Unit of Human Communication, Learning, and Development (HCLD), Faculty of Education, The University of Hong Kong, Hong Kong, Hong Kong SAR, China; ^2^Speech and Neuromodulation Laboratory, Academic Unit of Human Communication, Learning, and Development (HCLD), Faculty of Education, The University of Hong Kong, Hong Kong, Hong Kong SAR, China

**Keywords:** post-stroke aphasia, electroencephalography, transcranial alternating current stimulation, dysfunctome, oscillatory network, individualized neurorehabilitation

## Abstract

**Introduction:**

People with post-stroke aphasia (PSA) exhibit significant interindividual variability attributed to distinctive network disruption patterns across individuals. This complexity limits the effectiveness of conventional one-size-fits-all brain stimulation approaches, but to date no individualized tACS targeting on functional network was studied in PSA. This two-phase study aimed to investigate the immediate network-modulation and language-facilitation effects of dual-site in-phase tACS utilizing a novel individualized targeting method based on individual’s EEG dysfunctome.

**Methods:**

In the first phase, network-based linear regression was used to identify aphasia-severity-predictive dysfunctome from the speech-production EEG data of 15 Cantonese-speaking people with aphasia (PWA). Individualized stimulation targets were determined using two targeting principles. Restoration-based targeting aims to restore a target edge which is centralized within the target dysfunctome but weakly-connected in the individual, whereas enhancement-based targeting selects a strongly-connected target edge. The second phase involved a single-session double-blinded sham-controlled trial with the same group to evaluate the immediate effects of dual-site 7-Hz 1-mA tACS under four conditions: Restoration In-phase (RI), Enhancement In-phase (EI), Enhancement Anti-phase (EA), and Sham (SH).

**Results:**

In the first phase, we explored a range of frequency bands and EEG tasks and identified a left frontal-temporal theta network under divergent naming task that significantly predicted aphasia severity. The single-session clinical trial in the second phase demonstrated that RI condition produced increases in the target node strength, global network properties, and divergent naming performance, which were absent in sham and the other two real stimulation conditions.

**Discussion:**

This was the first-of-its-kind dysfunctome-based data-driven individualized tACS demonstrated immediate neuromodulatory effects in PSA. The findings suggest that EEG dysfunctome can help pinpointing effective individualized targets for tACS to promote clinically-beneficial functional reorganization. Despite limited generalizability due to the small sample, this methodology holds significant potential for application in longer-term treatment and other network-based disorders.

## Graphical Abstract

This two-phase study aimed to investigate the feasibility of using a novel EEG-guided, dysfunctome-based, data-driven targeting method to individualize tACS stimulation targets to promote neural- and language-facilitation in people with chronic post-stroke aphasia. Results support the feasibility of this approach by demonstrating personally-relevant target identification, safety, immediate network changes, and detectable language facilitation effect in restoration in-phase (RI) condition. This approach holds potential for multi-session stimulation in promoting long-term language recovery. 
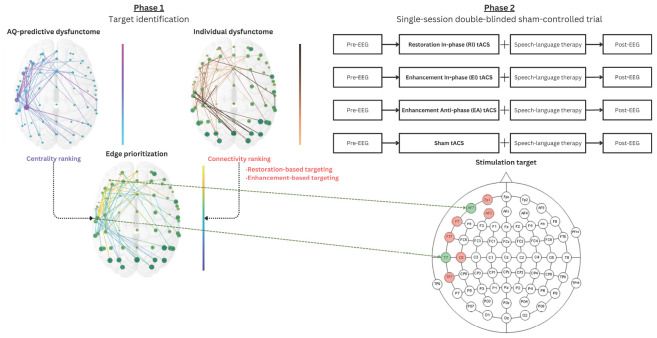

## 1 Introduction

Post-Stroke Aphasia (PSA) is a language disorder caused by damage to the left-hemisphere-dominant language processing network in the brain following a stroke (Ueno et al., 2011; Kümmerer et al., 2013; Geranmayeh et al., 2014; Fridriksson et al., 2016; Siegel et al., 2016; Hartwigsen and Saur, 2019). Due to the diverse network disruption patterns, people with aphasia (PWA) are considered to be a heterogenous group with individually varying language impairment profile in terms of severity, subtypes, and prognosis (Pedersen et al., 2004). Currently, speech-language-therapy (SLT) remains to be the mainstay of aphasia treatment. However, such behavioral-oriented approach has faced substantial challenges because of its limited efficacy and its inability to target the neurological underpinnings of the symptoms. To address this issue, non-invasive brain stimulation (NIBS) techniques have gained increasing attention as an adjunct treatment to boost the therapeutic effects of SLT (Williams et al., 2024). Today, NIBS in aphasia treatment is undergoing early proof-of-principle stage, yielding mixed results from various distinctive, or sometimes even contradicted stimulation principles (Miceli and Vogel-Eyny, 2016; Biou et al., 2019; Bucur and Papagno, 2019; Ding et al., 2022; Han et al., 2024). Although evidence of these techniques was generally positive, the emphasis of “individualization” was surprisingly low. Most existing studies employed one-size-fits-all stimulation protocols (Williams et al., 2024), which are deemed to be suboptimal for PWA because of the high inter-individual variability in their network disruption patterns after stroke (Shah-Basak et al., 2023). In other words, it is less likely for a single stimulation protocol focusing on a specific focal region to be able to bring universal effectiveness across PWA. This highlights the pressing need to develop individualized NIBS techniques for PSA.

There are fundamentally two types of approach in devising individualized NIBS techniques for PSA: the theory-driven approach and the data-driven approach. Theory-driven approaches select individualized stimulation targets based on established theories regarding how NIBS can facilitate recovery mechanism and assign individuals to particular pre-defined stimulation strategy based on certain criteria. However, recent findings suggested that following an extensive structure-function remapping process during stroke recovery, a distributed domain-general brain regions can be involved in the newly consolidated language network (Saur et al., 2006; Turkeltaub et al., 2011; Hartwigsen and Saur, 2019; Kiran et al., 2019). Apparently, restricting the stimulation targets to certain pre-defined regions can be potentially suboptimal for certain individuals. Alternatively, data-driven approaches offer several key advantages. First, they do not rely on generalized models that often reduce individuals’ unique characteristics to a winner-takes-all universal pattern, therefore, they allow tailoring treatment decisions based on actual patterns observed in the individual. Second, data-driven approaches excel at handling complexity, that is, when theoretical models fall short in capturing the intricate interactions of multiple contributing factors, data-driven approaches can leverage large datasets to identify patterns and uncover relationships that might not align with existing theories. Third, data-driven approaches provide the flexibility to adapt treatment decisions in response to dynamic changes in an individual’s condition.

Currently, there are several attempts in aphasia research that adopted the data-driven approach (e.g., Richardson et al., 2015). These studies mainly adopted two types of individualized target selection approaches: restoration-based targeting and enhancement-based targeting (Shah-Basak et al., 2023). Restoration-based targeting approaches aim to utilize NIBS techniques to normalize the aberrant activities, as compared to typical patterns observed in healthy individuals, identified in pre-intervention brain scan. While enhancement-based targeting approaches typically integrate functional imaging with language tasks to identify an individual’s activation patterns during the target behavior. These methods often select stimulation targets at the locations of maximal activation (Richardson et al., 2015; Ulm et al., 2015; Griffis et al., 2016; Fridriksson et al., 2018; Cherney et al., 2021). This approach aims at boosting an individual’s activation through NIBS promotes recovery, as the activation patterns are believed to represent the optimal neural pathways within the individual’s reorganized network.

To date, there is no agreement on the most effective individualization approach for aphasia treatment, and the existing studies also come with certain limitations. In particular, these studies focused on stimulation techniques aimed to modulate the level of excitation of neuronal activities at the localized brain regions (Brunoni et al., 2012; Klomjai et al., 2015; Roche et al., 2015), while overlooking the network dynamics rooting the problem. In fact, aphasia is widely-recognized as a “network disorder” (Kiran and Thompson, 2019; Kiran et al., 2019; Meier et al., 2019). This means that the impairment in language function is not solely attributable to dysfunction in isolated brain regions but rather to disrupted interactions between large-scale neural networks. By focusing only on localized neuromodulation, these approaches may fail to address the broader connectivity issues that underpin aphasia. A more comprehensive strategy would involve targeting the dysfunctional network dynamics, such as abnormal connectivity patterns, to restore the overall network balance.

**FIGURE 1:**
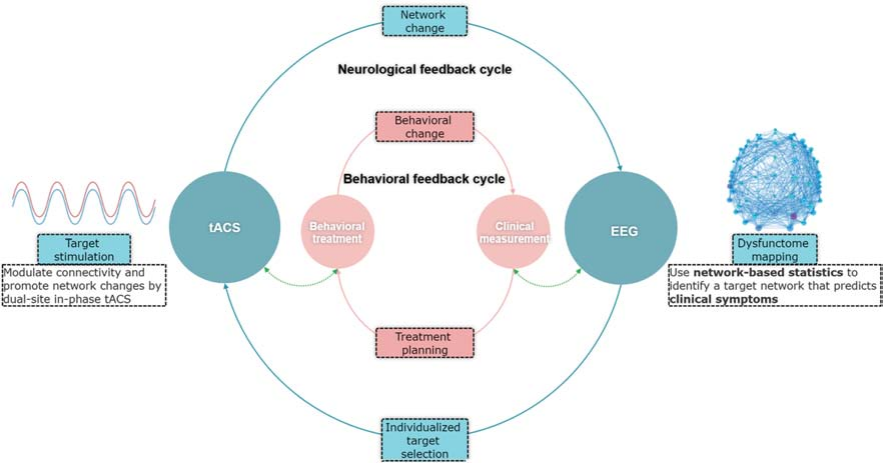
Conceptual framework of the EEG-guided individualized tACS based on dysfunctome mapping.

To address the above limitation, this study aimed to investigate the feasibility of using electroencephalography (EEG) dysfunctome guide individualized dual-site in-phase transcranial alternating current stimulation (tACS) for aphasia treatment. Dysfunctome is a comprehensive mapping of symptom-predictive network components in the brain-wide network (Horn, 2025). Fundamentally, we proposed a data-driven, individualized targeting approach embedded within a conceptualized neurological feedback cycle (Figure 1). This approach leverages individual’s dysfunctome to guide the selection of stimulation targets. The stimulation, in turn, is expected to induce changes in the individual’s dysfunctome, creating an adaptive neurological feedback cycle.

Specifically, dysfunctome mapping in EEG involves identification of symptom-predictive oscillatory network under a particular neural state. TACS is a stimulation technique used to modulate neuronal oscillatory activities non-invasively (Herrmann et al., 2013; Elyamany et al., 2021; Wischnewski et al., 2023). Research demonstrated that in-phase tACS can modulate functional connectivity through coupling the oscillatory activities between two stimulation sites by applying an identical, phase-aligned waveforms to both stimulation sites (Polanía et al., 2012; Helfrich et al., 2014). As EEG measures electric fields in the brain through scalp electrodes and tACS modulates the electric fields through the same medium, theoretically, in-phase tACS and functional network detected by EEG through phase-based connectivity measures are causally linked. Therefore, the combination of EEG-dysfunctome assessment and in-phase tACS holds several advantages. First, this approach aims at modulating functional network related to aphasic symptoms rather than solely targeting the excitability of localized regions, which may better account for the complex dynamics of language processing. Second, EEG phase-based connectivity measures and in-phase tACS have direct reciprocal causal relationships, which creates an ideal combination for data-driven individualized adaptive feedback cycle. Lastly, both technologies are relatively accessible, cost-effective, and user-friendly, making them more practical for routine clinical applications.

Aiming to examine the feasibility of this novel individualized targeting approach, we conducted a two-phase clinical trial. In the first phase, a group of PWA underwent an EEG scan while performing various language production tasks. An exploratory analysis using Network-based statistics (Zalesky et al., 2010) was performed aiming to identify a significant network component that predicts aphasia symptoms (i.e., the dysfunctome). We aimed to select an optimal single target edge from the target dysfunctome for each individual, using data-driven selection methods. Two individualized stimulation target selection methods were tested: restoration-based targeting prioritizes centralized-but-weakly-connected edge based on the individual’s dysfunctome profile, aiming for restoration, whereas enhancement-based targeting prioritizes centralized-and-strongly-connected edge, aiming to enhance them further. Consequently, each participant was assigned both a restoration-based target and an enhancement-based target to guide the dual-site in-phase tACS intervention. In the second phase, the same group of participants was included in a single-session double-blinded sham-controlled trial. We compared four stimulation conditions: (1) Restoration-based In-phase (RI), (2) Enhancement-based In-phase (EI), (3) Enhancement-based Anti-phase (EA), and (4) Sham (SH). EA and SH served as control conditions. Specifically, EA condition was examined to test whether anti-phase stimulation, an opposite stimulation technique of in-phase stimulation aimed to decouple the oscillatory activities between the target sites, can reduce the connectivity of the strong connection identified through enhancement-based targeting and produce opposite effect. Each condition involved a single intervention session consisted of tACS and concurrent SLT, accompanied by immediate pre- and post-intervention EEG and language assessments. These assessments aimed to evaluate whether the intervention could induce immediate improvements in both the target neural network and language performance.

## 2 Materials and methods

### 2.1 Materials

#### 2.1.1 Participants

In phase 1, a total of 15 Cantonese-speaking PWA (age: mean = 54.7, s.d. = 9.31; 5 females; Table 1) lived in Hong Kong were recruited to join the study. All participants were in the chronic stage of recovery (>6 months post-onset) following their first-ever stroke and were clinically diagnosed with aphasia using the Cantonese Aphasia Battery (Yiu, 1992) by a certified speech-language pathologist. All participants were right-handed verified by the Chinese version of Edinburgh Handedness Inventory (Yang et al., 2018). Exclusion criteria included aphasia due to causes other than stroke, a history of other developmental or acquired conditions affecting cognitive or language abilities, severe cognitive impairment, global aphasia, and any medical conditions contradicting transcranial electrical stimulation according to the safety guidelines (Antal et al., 2017). Written consent was obtained from all participants before the study began. This study was approved by the Human Research Ethics Committee of the University of Hong Kong [Approval Number: EA230112].

**TABLE 1:** Clinical features of participants.

ID	Gender	Age (y)	Education (y)	Post-onset (m)	Etiology	Lesion	Type of aphasia	Fluency	AQ
P004	M	61	16	22	Hemorrhagic	Left parietal, temporal lobes	Anomic	Fluent	85.9
P005	F	46	17	34	Ischemic	Left hemisphere	Broca	Non-fluent	71.4
P008	F	60	9	100	Unspecified	Left hemisphere	Anomic	Fluent	91.5
P009	M	59	11	39	Hemorrhagic	Left internal capsule	TM	Non-fluent	76.6
P010	M	49	11	47	Hemorrhagic	Left internal capsule	Anomic	Fluent	90.3
P011	M	57	13	8	Hemorrhagic	Left posterior parietal, temporal lobes	Wernicke	Fluent	35.9
P012	F	61	11	17	Ischemic	Left frontal, parietal, temporal lobes, BG	Broca	Non-fluent	52
P013	M	54	9	23	Hemorrhagic	Left putamen	Isolation	Non-fluent	23.9
P014	M	63	8	25	Ischemic	Left hemisphere	TM	Non-fluent	54.8
P016	M	66	20	13	Hemorrhagic	Left hemisphere	Anomic	Fluent	94.2
P017	M	50	11	10	Ischemic	Left parietal lobe	Wernicke	Fluent	40.9
P019	F	60	11	30	Hemorrhagic	Left putamen	TM	Non-fluent	57.5
P020	F	43	11	43	Hemorrhagic	Left hemisphere	Anomic	Fluent	81.1
P021	M	62	24	53	Hemorrhagic	Left frontal, parietal, temporal, occipital lobes	Isolation	Non-fluent	32.6
P022	M	31	16	6	Hemorrhagic	Left parietal, occipital lobes	TS	Fluent	63

BG, basal ganglia; TM, transcortical motor; TS, transcortical sensory; AQ, aphasia quotient.

In phase 2, all participants in phase 1 were invited to join the clinical trial. Three participants (P004, P017, P022) withdrew from this phase due to personal reasons. One participant (P009) withdrew from the study after completion of the first stimulation session due to experiencing considerable fatigue after the session. A total of 11 participants had completed the phase 2 study.

#### 2.1.2 EEG instruments

Electroencephalography recordings were collected using a BrainVision actiCHamp amplifier with 61 electrodes mounted in BrainVision actiCAP snap, following the extended International 10–20 positioning system (Oostenveld and Praamstra, 2001). Easycap HiCl Electrolyte-Gel was applied for electrode conductivity. To monitor physiological movements during speech that could introduce excessive artifacts into the EEG recordings, bipolar surface electromyograms (EMGs) were placed over the left superior and inferior orbicularis oris muscles to collect electromyographic signals (Lapatki et al., 2010) for subsequent speech production artifacts correction procedures (Porcaro et al., 2015). The online sampling rate was set to 512 Hz. Impedances were kept below 33 kΩ. A frequency range of 0.1–100 Hz was applied for online bandpass filtering. EEG and audio data were recorded while participants performed specific tasks.

#### 2.1.3 EEG tasks

Four EEG conditions were implemented including a resting state and three overt speech production tasks: (1) a delayed confrontation naming task, (2) a divergent naming task, and (3) a discourse production task.

##### 2.1.3.1 Resting-State

Participants were seated calmly in front of a computer screen 80 cm from their eyes and asked to focus on a central fixation point displayed on a white background on the screen. The task consisted of a 2-min eye-open scan followed by a 2-min eye-closed wakefulness scan, each prompted by spoken and written instructions.

##### 2.1.3.2 Delayed confrontation naming task

Participants were instructed to name an object shown as a line drawing on the computer screen as soon as they heard a sound signal, which occurred 4 s after the image appeared. Before the sound signal, participants were asked to simply view the image without responding. Each trial allowed a 20-s window for participants to respond. The next image was displayed only after the participant’s response, with the examiner manually controlling the presentation. The images used were taken from the Chinese version of the Boston Naming Test (Cheung et al., 2004). The task included 5 practice trials and 30 actual trials.

##### 2.1.3.3 Divergent naming task

Participants were asked to name as many single words as possible within a given semantic category. The categories included both high- (e.g., land animals) and low-frequency (e.g., birds) conditions and were prompted by spoken and written instructions. Participants were given 1 min to respond for each trial. The task consisted of 1 practice trial and 4 actual trials.

##### 2.1.3.4 Discourse production task

Participants were instructed to spontaneously produce monologues based on 7 discourse prompts selected from the Cantonese AphasiaBank (Kong and Law, 2019). These included two well-known narratives, two single-picture descriptions, two sequential-picture descriptions, and one procedural discourse. Participants were allowed unlimited time during the preparation stage to review the stimulus before beginning their monologue. Once the monologue began, a 3-min time limit was applied for each discourse task. All picture stimuli were displayed on the screen, with the examiner managing the timing of their presentation.

#### 2.1.4 TACS instruments

Transcranial alternating current stimulation was delivered by the Soterix Medical MxN-9 HD tES stimulator (Soterix Medical, New York, USA) through circular Ag/AgCl electrodes (1 cm radius, 5.99e7 S/m) attached to plastic hybrid holders that fixed both tES electrodes and EEG electrodes on the same EEG cap. The electrodes were filled with conductive gel (4 mm thickness, 1.4 S/m). Impedance of the tACS electrodes was kept equal to or below 10 kΩ.

### 2.2 Methods

#### 2.2.1 Phase 1: identification of target dysfunctome

##### 2.2.1.1 Design and procedure

All 15 participants from phase 1 underwent an EEG session utilizing the tasks and stimuli detailed in the “Section 2.1 Materials.” EEG data was preprocessed and segmented into time series for connectivity estimation. Network-based statistic (NBS) was applied to identify aphasia-related dysfunctomes. These results informed restoration-based and enhancement-based individualized stimulation targets for each participant, to be used in the phase 2 clinical trial.

##### 2.2.1.2 EEG preprocessing

All pre-processing procedures were performed using in-house routines and the EEGLAB toolbox (Delorme and Makeig, 2004; RRID:SCR_007292) on MATLAB version 9.14 (The MathWorks Inc, 2022; RRID:SCR_001622). The raw EEG signals were subjected to a bandpass filtering process with cut-off frequencies of 0.1 and 40 Hz. Bad channels were identified through visual inspection and corrected using spherical interpolation (Perrin et al., 1987). The data was then down-sampled to 250 Hz. To correct artifacts, an independent component analysis was conducted. Component classification was performed using the ICLabel plugin from EEGLAB, rejecting components labeled as “eye,” “muscle,” or “channel noise” with a probability greater than 50%. On top of that, we also rejected components showing higher-than-majority correlation with the EMG signals recorded from the lip muscles (Porcaro et al., 2015). The cleaned data were then reconstituted into channel space and underwent a final visual inspection to ensure quality. Subsequently, the signals were converted to an average reference. EEG signals were filtered into different frequency bands, including delta (1–4 Hz), theta (4–8 Hz), alpha (8–12 Hz), beta (15–30 Hz), and low-gamma (31–40 Hz) ranges. The EEG time series of each frequency band was then obtained by segmenting the signals into 4-s intervals. Time series with amplitude exceeding ±50 μV were rejected.

##### 2.2.1.3 Connectivity estimation

Connectivity matrices were constructed in sensor space, in which each electrode channel was defined as a node, and the connectivity between each pair of electrodes was defined as an edge. Phase Lag Index (PLI) was used as the connectivity estimator, which ranges from 0 to 1 and is robust against the effects of volume conduction (Peraza et al., 2012; Hardmeier et al., 2014). The calculation of the PLI was performed using in-house MATLAB code. A 61 × 61 connectivity matrix was constructed within each frequency band in each EEG task for each individual, resulting in a total of 20 sets connectivity matrices (5 frequency bands × 4 EEG tasks) for exploration in subsequent network-based statistical analyses.

##### 2.2.1.4 Network-based statistic

**FIGURE 2:**
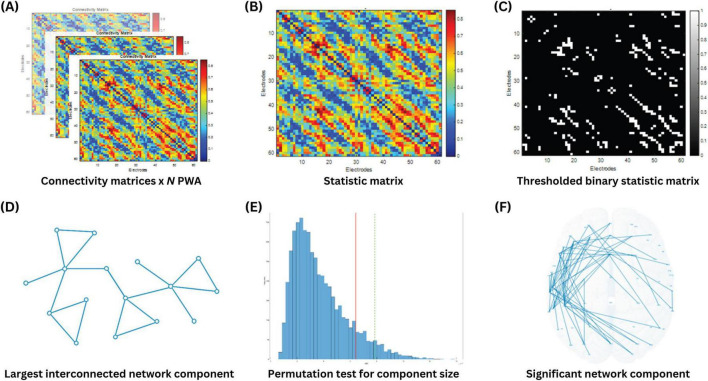
Identification of AQ-predictive dysfunctome using network-based statistics. **(A)** *N* connectivity matrices were obtained from *N* PWA. **(B)** Mass univariate linear regression was conducted for all edges to calculate a *t*-value for each edge in the statistic matrix, where higher *t*-values indicated a more significant positive relationship between the edge weight and aphasia quotient. **(C)** A *t*-value threshold (primary threshold) was applied to the statistic matrix to retain only the edges that showed the highest significance. **(D)** The size of the largest interconnected network component was extracted from the thresholded binary statistic matrix. **(E)** A permutation test was performed to obtain the random distribution of the largest network component size through 10000 iterations. **(F)** Statistical significance of the observed network component was determined.

All 20 connectivity matrices were included in the exploratory analysis using the Network-Based Statistic toolbox in MATLAB (Zalesky et al., 2010; RRID:SCR_002454). The goal of this analysis was to identify sub-networks comprising interconnected edges significantly predict aphasia severity (i.e., aphasia dysfunctome), as measured by aphasia quotient (AQ). As illustrated in Figure 2, the process began by performing mass univariate regression models on all edges to generate a single “statistic matrix,” where each element of the matrix represented the *t*-value of the corresponding edge. A primary threshold (a *t*-value threshold) was then applied to identify edges that showed a significant association with the AQ. This resulted in a binary statistic matrix, where edges exceeding the primary threshold were assigned a value of “1,” and all other edges were assigned a value of “0.”

Using this binary statistic matrix, network components were formed by linking all interconnected edges. The size of the largest network component was quantified by calculating the number of edges involved in the component. According to the assumptions of the NBS, cognitive processes rely on interconnected sub-networks serving as neural substrates, rather than on isolated connections distributed across the brain. Consequently, individual edges that were detached from the largest network component were considered less critical in supporting the cognitive process of interest.

Next, to evaluate the statistical significance of the observed network component, a permutation test was performed. In this test, the data labels were randomly shuffled across participants, and the size of the largest network component was recalculated for each permutation. This process was repeated 10,000 times to generate a null distribution of component sizes that could arise by chance. The observed network component was deemed statistically significant if its size exceeded the 95th percentile of the null distribution, corresponding to a *p*-value below 0.05.

The choice of primary threshold is considered arbitrary and open to exploration. In particular, applying a higher primary threshold is expected to extract smaller network components, and vice versa. There is no definitive rule for selecting primary threshold, as different cognitive processes may involve varying degree of focality in their neural substrates. A stringent threshold is appropriate when the effect is expected to be strong but localized, whereas a less stringent threshold is better suited for detecting weaker effects that are distributed across broader regions. Since predicting the nature of these effects is inherently challenging, analyzing the data with a range of thresholds allowed for greater flexibility and insight into the network patterns (Fornito et al., 2016; Mehraram et al., 2023). Therefore, the above-described procedure was repeated with multiple primary thresholds, starting from 2 to 4 with increments of 0.2 per step.

Finally, we evaluated which significant network component identified via NBS had the highest correlation with AQ. For this purpose, the individual averaged connectivity within each significant network component was calculated and entered into a Pearson’s correlation analysis with AQ. The network component with the highest correlation coefficient was selected as the target dysfunctome.

##### 2.2.1.5 Dysfunctome-based individualized targeting

**FIGURE 3:**
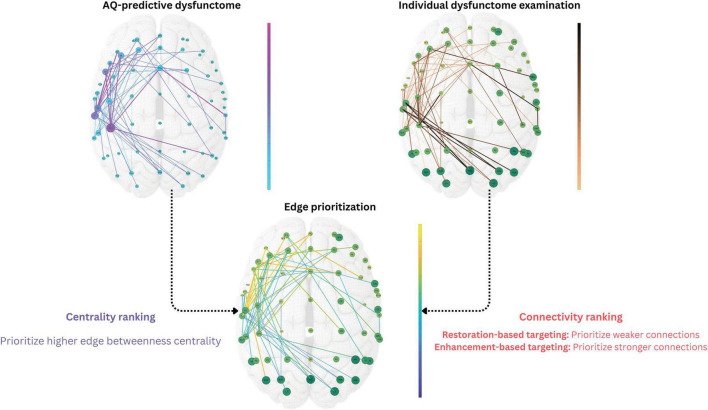
Dysfunctome-based individualized targeting method. The edge betweenness centrality was calculated for each edge within the target dysfunctome, a centrality ranking was obtained and normalized to a 0-to-1 value for each edge. The edge weight of the individual’s dysfunctome profile within the target dysfunctome was used to estimate the connectivity ranking for each edge, which was also normalized to a 0-to-1 value. In restoration-based targeting, edges with weaker connectivity were ranked higher, whereas in enhancement-based targeting, edges with stronger connectivity were ranked higher instead. Priority index was the average of the values of two rankings to ensure equal contribution of two factors. The single edge with the highest priority index was selected as the stimulation target for the individual where two stimulating electrodes were placed in the dual-site tACS.

The dysfunctome-based individualized targeting consists of three steps: (1) dysfunctome identification, (2) individual dysfunctome examination, and (3) edge prioritization (Figure 3). This method requires two input information, one is the target dysfunctome defined as a binary matrix derived from the network-based linear regression, another is the individual’s dysfunctome profile defined as a weighted matrix. This method outputs a single edge which serves as the stimulation target for dual-site in-phase tACS with the stimulating electrodes positioned at the two nodes connected by the target edge.

After the exploratory analysis in phase 1, we identified an aphasia-predictive theta dysfunctome during divergent-naming task (see Section “3 Results” for details). The primary goal of the stimulation was to enhance the connectivity within this target dysfunctome. Given that the mechanism of dual-site in-phase tACS is to increase the connectivity between two stimulation sites, the individualized stimulation target was defined as a single edge within the target dysfunctome that was both “topologically important” and “individually relevant” based on two key factors: *edge betweenness centrality* and *connectivity*.

Edge betweenness centrality defines how an edge is considered to be topologically important based on the structure of the network. Particularly, it quantifies the importance of an edge based on the proportion of “shortest paths” that pass through it (Girvan and Newman, 2002). Edges with higher betweenness centrality are considered “bottlenecks” in the network which play a critical role in the flow of information of the network (Freeman, 1977). Edges with higher edge betweenness centrality in the target dysfunctome were prioritized for stimulation.

To determine which edges are individually relevant, the connectivity of the edges within an individual dysfunctome profile was considered. As discussed, it is indefinitive about whether stimulation should aim for restoring weak connections that were damaged from the stroke or further enhancing strong connections that showed functional importance in the reorganized network, therefore, two distinct targets were chosen based on connectivity. For restoration-based targeting, edges with lower connectivity were prioritized, while for enhancement-based targeting, edges with higher connectivity were prioritized.

To equally weigh the influence of both edge betweenness centrality and connectivity in the edge prioritization process, all edges within the target dysfunctome were ranked and normalized based on these two variables. This process resulted in a 0-to-1 value for both *centrality ranking* and *connectivity ranking* for each edge within the target dysfunctome. By averaging these two rankings, a *priority index* ranging from 0 to 1 was obtained for each edge, representing its priority for stimulation. The edge with the highest priority index was selected as the target for the dual-site in-phase tACS.

#### 2.2.2 Phase 2: single-session clinical trial

##### 2.2.2.1 Design and procedure

**FIGURE 4:**
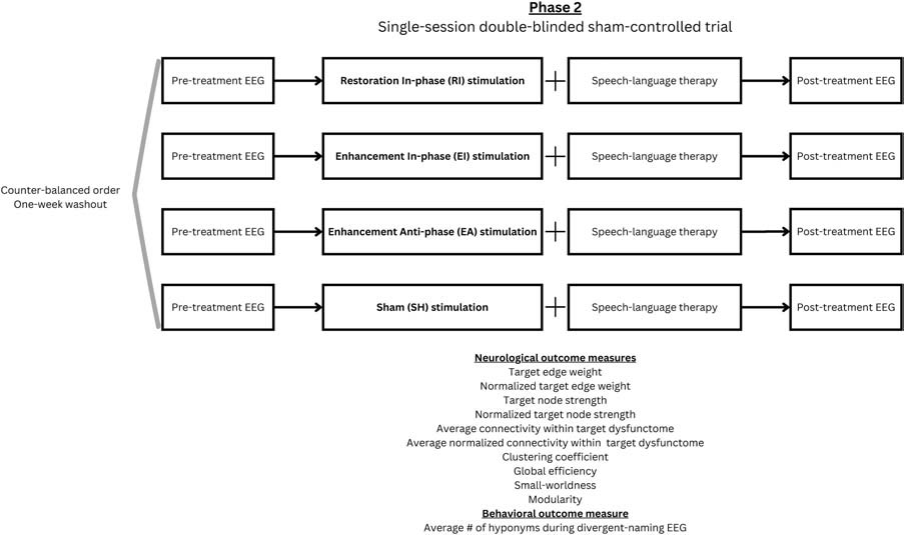
Phase 2 experimental design.

In phase 2, a single-session double-blinded sham-controlled trial was conducted with procedure illustrated in Figure 4. All participants underwent four 30-minute tACS stimulation sessions, with each session corresponding to one of the following stimulation conditions: (1) Restoration-based In-phase (RI), (2) Enhancement-based In-phase (EI), (3) Enhancement-based Anti-phase (EA), and (4) Sham (SH). During each session, a concurrent 30-min SLT was conducted by trained student clinicians. All student clinicians and participants were blinded to the stimulation condition. A 1-week washout period was implemented between sessions to minimize carryover effects, and the order of stimulation conditions was counterbalanced across participants. Five trained speech therapy students blinded to the experimental condition were responsible for carrying out all assessment, scoring, and the SLT. The first author was responsible for operating the stimulation machine and was not involved in the SLT and any outcome scoring.

Within each session, EEG scans were implemented immediately before and after stimulation to assess immediate effects of the intervention. The procedure and analytic method of EEG were consistent with those used in phase 1 but included only the divergent naming task (which derived the target dysfunctome). The same set of items was used for both pre- and post-intervention assessments within a session. To reduce practice effects, two distinct sets of items were alternated across sessions. After each session, a questionnaire was given to obtain participant’s subjective sensation, perception about being stimulated and adverse events associated with the stimulation. This questionnaire was adapted from the recommended template provided in the tES safety guidelines (Antal et al., 2017).

##### 2.2.2.2 TACS protocol

In all conditions, a dual-site 3 × 1 HD electrode montage was employed. This setup involved two stimulating electrodes positioned at the target nodes required for synchronization (i.e., the target edge), while three return electrodes were placed at the nearest channels surrounded each stimulating electrode (based on the configuration of the 64-channel EEG cap) to create a focal electrical field (Helfrich et al., 2014; Keator, 2022). The current intensity was set to 0.5 mA for both stimulating electrodes, and 0.17 mA for all returning electrodes, which had opposite polarity to the stimulating electrode they surrounded. In the in-phase conditions, both stimulating electrodes were set to the same polarity, whereas in the anti-phase condition, the two stimulating electrodes were assigned opposite polarities.

As we identified a theta (4–8 Hz) target dysfunctome in phase 1, the stimulation frequency for all conditions was set to 7 Hz. This frequency was chosen because previous studies suggested that a higher frontal-centric theta peak is associated with greater performance in cognitive tasks among healthy individuals (Moran et al., 2010).

The stimulation target of the sham condition followed enhancement-based target. During sham stimulation, the stimulator ramped up to the target intensity within the first 30 s of the session to mimic the stimulation sensation and then immediately ramped down to a negligible intensity for the remainder of the stimulation period. This procedure was repeated during the final minute of the session. This “fade-in, short-stimulation, fade-out” (FSF) protocol is the most commonly-used sham procedure across clinical trials and is considered effective for blinding when the stimulation intensity does not exceed 1 mA (Ambrus et al., 2012).

##### 2.2.2.3 Speech language therapy

The 30-min SLT focused on speech production training. A two-phase repetitive training routine was conducted. The first phase consisted of a cueing-hierarchy naming training (McNeil et al., 1995), which utilized a series of increasingly less explicit cues to help the participant retrieve the correct name of an object depicted in a picture. Once the participant demonstrated retention of the trained target words, the training transitioned to the second part of the routine, which utilized the Response Elaboration Training (RET) (Kearns, 1985) approach. In this phase, the therapist asked the participant to describe a realistic, action-depicting picture that included a few target words trained in the first phase. Following the participant’s initial verbal response, the therapist provided a verbal model and asked follow-up wh-questions to prompt an expanded version of the sentence. This process was repeated multiple times until the participant was unable to further elaborate. Through this iterative method, the participant’s speech was shaped to become more detailed and complex.

All SLT stimuli were obtained from open-source picture banks available online. The target words were selected based on the individual participant’s ability level. One week prior to the stimulation sessions, a pre-treatment probe of 140 key words was administered to each participant to identify the most appropriate training items. Priority was given to words that the participant initially had difficulty producing but could successfully retrieve with cueing.

##### 2.2.2.4 Outcome measures

To investigate the effects of the stimulation on the target dysfunctome, the primary neurological outcome measures included the (1) connectivity of the target edge (estimated by phase lag index), (2) target node strength (calculated as the average node strength of the two nodes connected by the target edge), and (3) average connectivity within the target dysfunctome. Additionally, to determine whether the stimulation effects specifically modulated the target connections without influencing other connections, changes in the normalized connectivity of the target edge, normalized target node strength, and the average normalized connectivity within the target dysfunctome were also analyzed.

In addition to the connectivity changes within the target dysfunctome, we also explored the changes in global network properties during the divergent-naming EEG (the task-dependent EEG that derived the target dysfunctome). This included clustering coefficients, global efficiency, small-worldness, and modularity. These global network metrics represent important characteristics of the brain’s overall network organization and efficiency (Bullmore and Sporns, 2009; Ismail and Karwowski, 2020). The clustering coefficient reflects the tendency of nodes to form tightly connected groups, which is indicative of local specialization (Kaiser, 2008). Global efficiency measures the brain’s ability to integrate information across distant regions, serving as a marker of functional integration (Bullmore and Sporns, 2012). Small-worldness captures the balance between local specialization and global integration, a hallmark of efficient brain networks (Bullmore and Sporns, 2012). While modularity quantifies the degree to which the network is organized into distinct communities or modules that support specialized processing (Newman and Girvan, 2004). By examining these network properties, we aimed to assess whether the treatment not only influenced the target dysfunctome but also induced broader changes in the brain’s functional organization during a language task. All global network metrics were evaluated in unweighted connectivity matrix with a density threshold of 35% as binarization criteria. This threshold was determined based on prior exploratory analysis of multiple density thresholds ranging from 5% to 50% with 5% increments. The density threshold of 35% captured the most significant post-treatment differences in global network metrics across conditions. These measures were calculated by Brain Connectivity Toolbox (Rubinov and Sporns, 2010; RRID:SCR_004841) in MATLAB.

Apart from neurological changes, it was also important to investigate whether changes in the neural network corresponded to language facilitation. To this end, we measured the average number of hyponyms generated per minute performed during the divergent-naming EEG.

### 2.3 Statistical analysis

Statistical analysis program JASP (JASP Team, 2024 version 0.19.3, Netherlands, RRID:SCR_015823) was used to implement Bayesian analysis. A series of Bayesian paired-samples *t*-tests were performed to evaluate the evidence for post-treatment changes in each outcome measure within each stimulation condition. Cauchy prior distribution with a scale parameter of 0.707, centered at zero, was employed, reflecting the assumption that the null and alternative hypotheses are equally likely to account for the data (van Ravenzwaaij and Etz, 2021). Sequential analysis was performed to monitor how the evidence supporting either the null or alternative hypothesis develops over the course of data collection. If the Bayes factor BF_10_ rapidly increases and stabilizes above 3, it indicates accumulating and substantial evidence in favor of the alternative hypothesis. Conversely, if the Bayes factor decreases and stabilizes below 1/3, it suggests substantial evidence for the null hypothesis. To compare post-treatment change values for each outcome measure across stimulation conditions, a Bayesian one-way repeated measures ANOVA was performed using the multivariate Cauchy prior for effect size estimation, followed by Bayesian *post hoc* pairwise comparisons between conditions. Finally, Bayesian correlation analyses with a stretched beta prior width of 1 were used to assess the strength of association between change values in neurological outcomes and change values in the language outcome.

## 3 Results

### 3.1 Phase 1: target dysfunctome

Within the 20 conditions (5 frequency bands × 4 EEG tasks) which underwent NBS exploration for identification of significant network components that positively predicted AQ, only three conditions showed significant network components with *p*-value below 0.05, they were resting-state alpha, resting-state beta, and divergent-naming theta networks. As multiple primary thresholds were used during the exploration, multiple network components within the same condition were found significant with different component sizes. Higher primary thresholds have the advantage of yielding smaller network components but often sacrifice meaningful network structures that reflect the coordinated activity of multiple regions supporting a cognitive function, the loss of network structure can diminish the detectability of important network hubs within the network component. To address this, for each condition, we selected the significant component generated at the highest primary threshold that still contained at least 3% of total edges (55 edges), ensuring the preservation of meaningful network structure.

**FIGURE 5:**
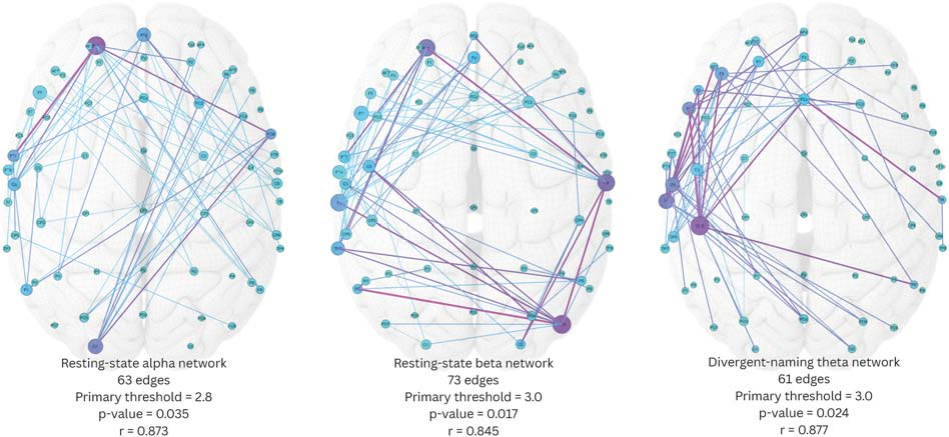
Three significant network components identified by network-based statistics. Edge color scales with edge betweenness centrality, node color scales with node betweenness centrality, node size scales with node degree. Pearson’s *r* indicates the correlation between the average connectivity within the network component and aphasia quotient.

The three resulting significant network components were shown in Figure 5. The resting-state alpha network had 63 edges, yielded from a primary threshold of 2.8 (*p* = 0.035). This alpha network had wide-spread edges connecting left temporal and left frontal regions, left frontal and right temporal regions, and right frontal and left occipital regions. Fp1 was identified as one of the most important network hubs with the highest node degree and node betweenness centrality. The resting-state beta network had 73 edges, yielded from a primary threshold of 3.0 (*p* = 0.017). The beta network had a round-shape circuit of edges connecting left temporal and left frontal regions, left frontal and right temporal regions, right temporal and right occipital regions, and right occipital back to left temporal regions. Within this network, T7, Fp1, C6, and PO8 were the four most important network hubs that showed higher node degrees and betweenness centralities. Lastly, the divergent-naming theta network had 61 edges, yielded from a primary threshold of 3.0 (*p* = 0.024). This network had densely interconnected edges within the left frontal-temporal regions, with several extended connections to the right temporal, parietal, and occipital regions, where CP3 was the most important network hub with the highest node degree and betweenness centrality. Notably, the divergent naming task possibly shifted the aphasia-related network activities from faster waves to a slower wave, and from widespread interregional activities to left-frontal-temporal focused activities.

We then estimated the Pearson correlation between the average connectivity of each of the three network components and AQ. The analysis revealed that the average connectivity of all three network components was significantly and positively correlated with AQ (resting-state alpha network: *r*(13) = 0.873, *p* < 0.001; resting-state beta network: *r*(13) = 0.845, *p* < 0.001; divergent-naming theta network: *r*(13) = 0.877, *p* < 0.001).

Among these, the divergent-naming theta network demonstrated the strongest correlation with AQ and was considered more representative of a language-specific network compared to the resting-state networks. Therefore, it was chosen as the target dysfunctome for subsequent stimulation in phase 2.

### 3.2 Phase 2: Single-session clinical trial

#### 3.2.1 Participant perception and safety

According to the post-stimulation questionnaire, all participants perceived that they had received real stimulation in all conditions including the sham condition, which ensured equal placebo effect across conditions. No serious adverse events were reported. The most frequently reported subjective sensations were mild tingling, mild itchiness at the stimulation sites, occurring 27.3% and 25% of the time, respectively. Fatigue following the session was reported 22.7% of time. On rare occasions, participants also experienced mild headaches (6.8%) and mild burning sensation at the stimulation sites (2.3%). Overall, the data indicated that the stimulation was safe.

#### 3.2.2 Individualized targets

**FIGURE 6:**
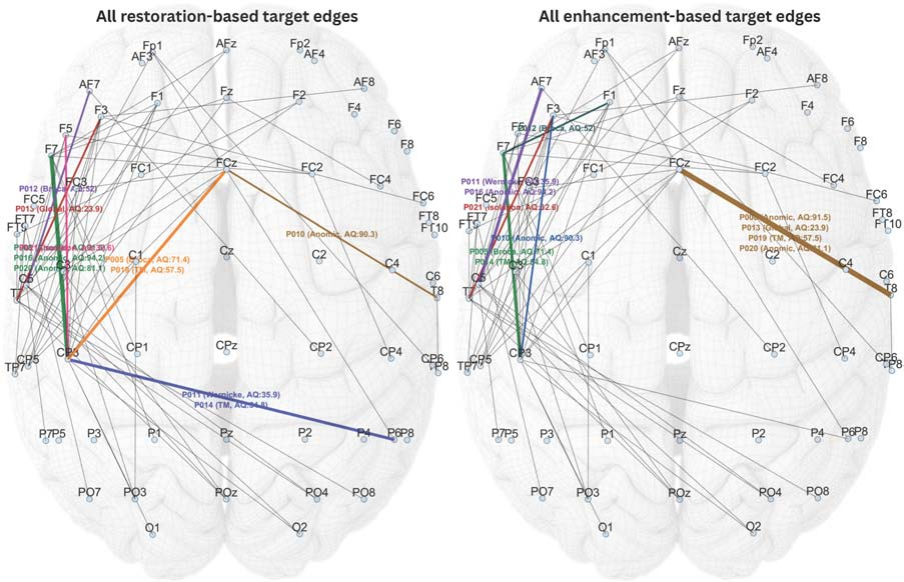
All individualized stimulation targets selected based on restoration-based and enhancement-based targeting. Different target edges are highlighted in different colors.

Figure 6 showed the selected stimulation targets for all participants based on both restoration-based and enhancement-based targeting approaches. In some cases, the edge with the highest priority index could not be selected as stimulation target because the corresponding stimulation sites were positioned too close to one another, which could prevent dual-site in-phase tACS from achieving the intended effect. In such instances, the next highest-priority edge that did not violate this criterion was chosen.

Given the wide range of participant characteristics in this study, including aphasia type, severity, and post-onset period, the proposed individualized targeting method appropriately selected different stimulation targets for each individual, as expected. Noteworthily, the targets selected through restoration-based targeting for some of the participants overlapped with the targets selected through enhancement-based targeting for other participants. This was considered normal, as individual dysfunctome profiles can vary across individuals. Nevertheless, it is not possible for the two selection methods to produce overlapping targets within the same individual.

#### 3.2.3 Post-treatment changes

**FIGURE 7:**
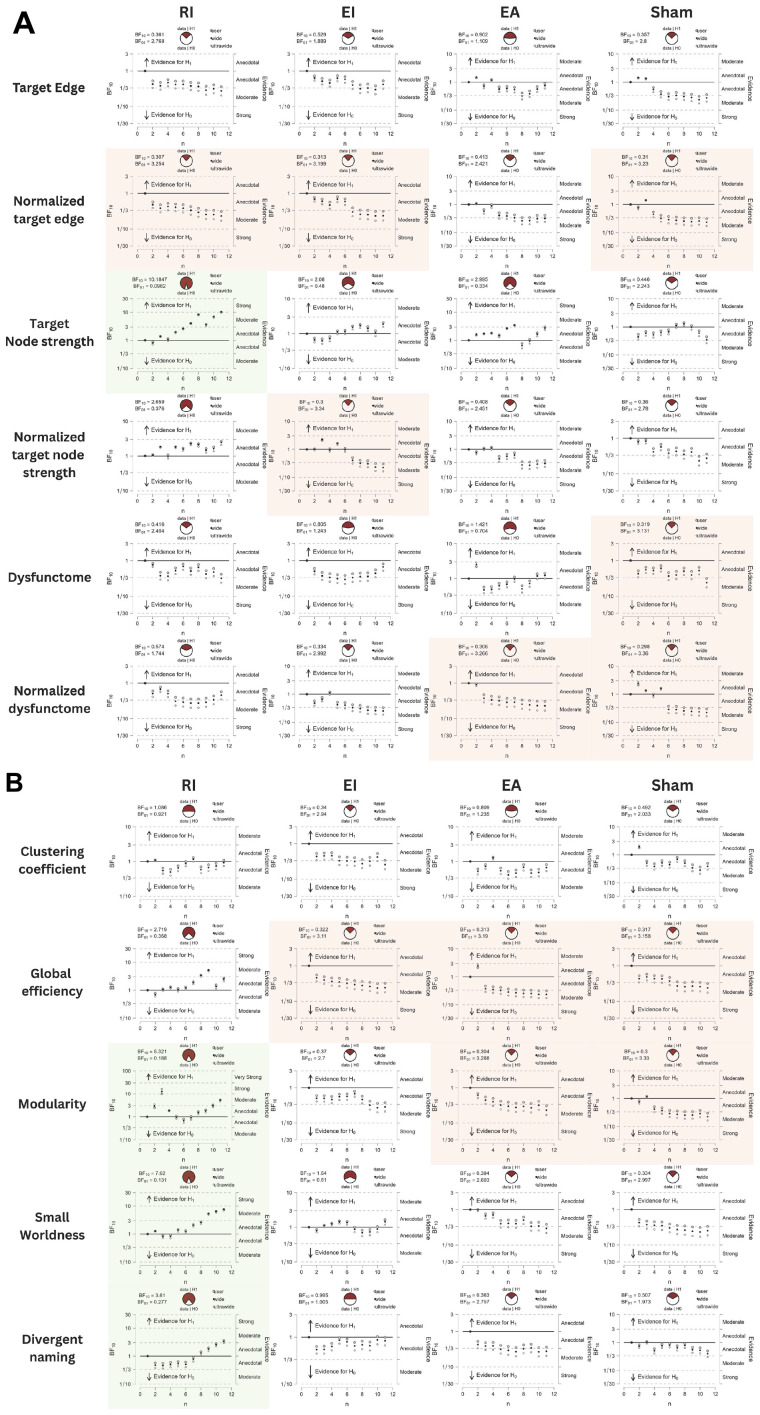
**(A,B)** Sequential analysis plots of Bayesian paired-sample *t*-tests of all outcomes. Variables with BF_10_ > 3 are highlighted in green, indicating substantial evidence favoring alternative hypothesis. Variables with BF_01_ > 3 are highlighted in red, indicating substantial evidence favoring null hypothesis.

Table 2 displayed the descriptive statistics of all pre- and post-test values of all conditions. The Bayesian statistic table (Table 3) and the sequential analysis plots (Figure 7) highlighted the evidence of post-treatment changes across all outcome measures evaluated by Bayesian paired-sample *t*-tests in each condition.

**TABLE 2:** Descriptive statistics of pre- and post-tests values of all outcome measures.

Outcomes	Restoration in-phase		Enhancement in-phase		Enhancement anti-phase		Sham	
	Pre	Post	Pre	Post	Pre	Post	Pre	Post
Target edge weight	0.4172 (0.3592)	0.5426 (0.4240)	0.5590 (0.3353)	0.5279 (0.2813)	0.5106 (0.3585)	0.6084 (0.1927)	0.4966 (0.2440)	0.4844 (0.3996)
Normalized target edge weight	−0.10 (1.94)	0.55 (2.06)	0.65 (1.61)	0.38 (1.35)	0.23 (1.58)	0.25 (1.01)	0.28 (0.87)	0.15 (1.72)
Target node strength	26.45 (4.85)	28.26 (4.12)	27.03 (1.85)	28.57 (3.95)	26.09 (3.95)	28.33 (3.67)	27.36 (5.46)	27.35 (5.04)
Normalized target node strength	−0.17 (0.50)	0.04 (0.34)	0.09 (0.88)	0.14 (0.50)	−0.05 (1.18)	0.28 (0.55)	−0.01 (0.56)	0.26 (0.94)
Connectivity in the target dysfunctome	0.5218 (0.1461)	0.5376 (0.1318)	0.5057 (0.1431)	0.5297 (0.1428)	0.5114 (0.1801)	0.5419 (0.1807)	0.5375 (0.0905)	0.5456 (0.1907)
Normalized connectivity in the target dysfunctome	0.41 (0.39)	0.29 (0.24)	0.30 (0.24)	0.33 (0.24)	0.26 (0.46)	0.28 (0.40)	0.37 (0.42)	0.41 (0.53)
Clustering coefficient	0.3965 (0.0274)	0.4117 (0.0292)	0.3968 (0.0414)	0.4072 (0.0179)	0.3899 (0.0159)	0.4088 (0.0317)	0.3991 (0.0216)	0.3985 (0.0315)
Global efficiency	0.6715 (0.0026)	0.6721 (0.0017)	0.6711 (0.0028)	0.6728 (0.0025)	0.6719 (0.0028)	0.6730 (0.0035)	0.6723 (0.0015)	0.6728 (0.0042)
Small-worldness	0.9057 (0.0823)	0.9838 (0.1423)	0.9421 (0.1661)	0.9910 (0.1101)	0.9540 (0.1574)	0.9968 (0.1173)	0.9617 (0.1629)	1.0068 (0.1500)
Modularity	0.1510 (0.0341)	0.1691 (0.0294)	0.1629 (0.0378)	0.1627 (0.0342)	0.1636 (0.0510)	0.1652 (0.0222)	0.1676 (0.0383)	0.1707 (0.0389)
# of hyponyms in divergent naming	3.25 (5.50)	4.00 (6.25)	3.25 (6.50)	4.25 (7.75)	3.25 (6.50)	2.75 (6.75)	1.75 (6.00)	1.75 (8.00)

Median (interquartile range), *N* = 11. Normalized scores = within-subject z-scores.

**TABLE 3:** Bayesian paired-sample *t*-tests examining significance of post-treatment change across conditions and outcomes.

	BF10 [95% CI of effect size]			
Post – pre	RI	EI	EA	Sham
Target edge	0.361 [−0.363, 0.712]	0.529 [−0.249, 0.866]	0.902 [−0.142, 1.030]	0.357 [−0.368, 0.706]
Normalized target edge	0.307* [−**0.459, 0.600]**	0.313* [−**0.443, 0.618]**	0.413 [−0.316, 0.774]	0.310* [−**0.452, 0.609]**
Target node strength	10.185** [0.203, 1.680]	2.084 [−0.012, 1.258]	2.995 [0.039, 1.354]	0.446 [−0.293, 0.804]
Normalized target node strength	2.659 [0.023, 1.322]	0.300* [−**0.495, 0.562]**	0.408 [−0.320, 0.768]	0.360 [−0.365, 0.710]
Dysfunctome	0.416 [−0.313, 0.776]	0.805 [−0.163, 0.997]	1.421 [−0.068, 1.156]	0.319* [−**0.427, 0.636]**
Normalized dysfunctome	0.574 [−0.893, 0.231]	0.334 [−0.668, 0.399]	0.306* [−**0.596, 0.463]**	0.298* [−**0.522, 0.534]**
Clustering coefficient	1.086 [−0.111, 1.082]	0.340 [−0.391, 0.679]	0.809 [−0.162, 0.999]	0.492 [−0.840, 0.267]
Global efficiency	2.719 [0.026, 1.328]	0.322* [−**0.422, 0.642]**	0.313* [−**0.620, 0.441]**	0.317* [−**0.629, 0.433]**
Modularity	5.321* [0.117, 1.505]	0.370 [−0.354, 0.724]	0.304* [−0.471, 0.588]	0.300* [−0.567, 0.490]
Small worldness	7.620* [0.165, 1.601]	1.640 [−0.047, 1.194]	0.384 [−0.340, 0.742]	0.334 [−0.400, 0.667]
Divergent naming	3.610* [0.065, 1.403]	0.995 [−0.126, 1.058]	0.363 [−0.362, 0.714]	0.507 [−0.259, 0.851]

*Moderate evidence with BF_10_ > 3 or <1/3, **Strong evidence with BF_10_ > 10 or <1/10, RI, restoration in-phase; EI, enhancement in-phase; EA, enhancement anti-phase.

Firstly, sham stimulation did not yield BF_10_ higher than 3 in any outcome while yielding BF_10_ below 1/3 in several outcomes including normalized target edge, target dysfunctome, normalized connectivity in target dysfunctome, global efficiency, and modularity. These results indicate that the data did not favor the alternative hypothesis across all neurological or language outcomes. This suggests that providing SLT alone with the placebo effect of stimulation was insufficient to induce notable neural or language change following a 30-min single-session dosage.

Among the three real stimulation conditions, RI condition was the only condition yielding moderate evidence of treatment effects across multiple neurological and language outcomes. This included target node strength (BF_10_ = 10.1847, posterior median = 0.906, 95% CI [0.203, 1.680]), modularity (BF_10_ = 5.321, posterior median = 0.776, 95% CI [0.117, 1.505]), small-worldness (BF_10_ = 7.62, posterior median = 0.848, 95% CI [0.165, 1.601]), and divergent naming performance (BF_10_ = 3.61, posterior median = 0.700, 95% CI [0.065, 1.403]). Sequential analyses showed robustness in target node strength and small-worldness as they demonstrated stability in BF_10_ upon accumulating data. The close-to-moderate evidence in increasing global efficiency (BF_10_ = 2.719, posterior median = 0.644, 95% CI [0.026, 1.328]) is also worth noting.

While EI and EA conditions did not yield substantial evidence of treatment effect in any outcomes, they still showed close-to-moderate evidence in increasing target node strength (EI: BF_10_ = 2.080, posterior median = 0.591, 95% CI [−0.012, 1.258]; EA: BF_10_ = 2.995, posterior median = 0.663, 95% CI [0.039, 1.354]). Another notable finding is the anecdotal evidence suggesting that the EA condition may increase connectivity within the target dysfunctome. While this effect did not demonstrate stability, it was the only condition for which the evidence favored the alternative hypothesis.

Overall, these findings indicate that RI stimulation was capable of increasing target node strength, but not the connectivity of the target edge nor the target dysfunctome. These effects reflect an increase in the overall connectivity of the target sites with a broader range of brain regions instead of increases limited within the dysfunctome. Since RI stimulation had the most substantial effect in increasing target node strength, probably due to the lower baseline values of the targets, this suggests a heightened centrality within the network of both target sites. Remarkably, RI stimulation also demonstrated a superior effect in improving global network properties, including global efficiency, modularity, and small-worldness. This indicated that RI stimulation may have a more pronounced impact on enhancing the global network change. The observed improvements in global efficiency suggested that RI stimulation facilitates better integration of information across distant brain regions. While enhanced modularity indicated that RI stimulation promotes more distinct community structures within the network, which may support specialized processing. Both the above properties may contribute to the increase in small-worldness reflecting an optimized balance between local specialization and global integration. These findings highlighted the potential of RI stimulation to induce widespread changes in brain connectivity beyond the target dysfunctome.

**FIGURE 8:**
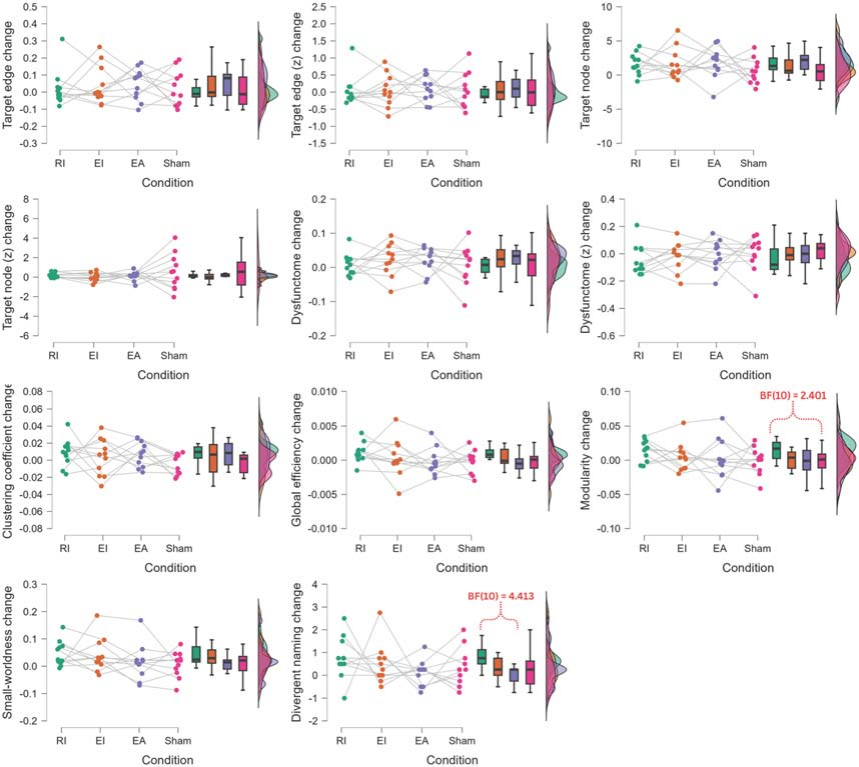
Bayesian one-way repeated-measure ANOVA of post-treatment change comparison across conditions. RI, restoration in-phase; EI, enhancement in-phase; EA, enhancement anti-phase.

Additional to the separate evaluations of post-treatment improvement for each of the conditions, a series of Bayesian one-way repeated-measure ANOVA were performed to compare the post-treatment change values across the four conditions for each outcome measure (Figure 8). Possibly due to the small sample size, no significant results were found. Nonetheless, Bayesian *post hoc* pairwise comparison found that RI condition had moderate evidence of larger post-treatment increase in divergent naming performance than EA condition (BF_10_ = 4.413), and close-to-moderate evidence of larger post-treatment increase in modularity than sham condition (BF_10_ = 2.401)

Lastly, Bayesian Pearson’s correlations were performed to investigate relationship between changes in different outcome variables observed in RI condition (Table 4). There is anecdotal evidence supporting that the change in divergent naming is negatively correlated with the change in normalized connectivity in the target dysfunctome (*r* = −0.503, BF_10_ = 1.123). Notably, there is moderate evidence supporting that the change in normalized connectivity in the target dysfunctome is also negatively correlated with the change in modularity (*r* = −0.677, BF_10_ = 3.781). Although preliminary, these findings suggest that a decrease in the connectivity in the target dysfunctome relative to the rest of the brain network may be associated with an increase in modularity and improvement in language performance.

**TABLE 4:** Bayesian correlation between post-treatment change values of outcome measures in RI condition.

Variables	Variables	Pearson’s *r*	BF10
Δ Divergent naming	Δ Normalized dysfunctome	−0.503	1.123
Δ Target edge	Δ Normalized target edge	0.982	64415.199
Δ Target edge	Δ Target node strength	0.658	3.183
Δ Target edge	Δ Normalized target node strength	0.532	1.313
Δ Target edge	Δ Dysfunctome	0.875	83.498
Δ Target edge	Δ Normalized dysfunctome	0.754	8.834
Δ Normalized target edge	Δ Target node strength	0.585	1.828
Δ Normalized target edge	Δ Normalized target node strength	0.589	1.877
Δ Normalized target edge	Δ Dysfunctome	0.806	18.962
Δ Normalized target edge	Δ Normalized dysfunctome	0.787	14.132
Δ Normalized target edge	Δ Global efficiency	0.512	1.178
Δ Target node strength	Δ Normalized target node strength	0.738	7.230
Δ Target node strength	Δ Dysfunctome	0.662	3.313
Δ Normalized target node strength	Δ Clustering coefficient	0.595	1.948
Δ Normalized target node strength	Δ Small worldness	0.532	1.317
Δ Dysfunctome	Δ Normalized dysfunctome	0.765	10.191
Δ Normalized dysfunctome	Δ Modularity	−0.677	3.781
Δ Clustering coefficient	Δ Small worldness	0.757	9.181

Only correlations with evidence favored alternative hypothesis are displayed.

## 4 Discussion

This two-phase study aimed to investigate the therapeutic effects of a single-session individualized dual-site in-phase tACS by utilizing a novel dysfunctome-based, data-driven method to select individualized stimulation target for PWA. In the first phase, we successfully identified an AQ-predictive theta dysfunctome from the task-dependent EEG data of a group of PWA performing divergent naming task. Based on the proposed dysfunctome-based individualized targeting method, we selected two individualized stimulation targets for each individual, one based on restoration principle, one based on enhancement principle. Particularly, restoration-based targeting selects a centralized edge within the target dysfunctome which is weakly-connected within the individual’s dysfunctome profile, whereas enhancement-based targeting selects a strongly-connected target edge instead. We observed that both selection principles successfully identified different target edges for participants with varying clinical profiles. This reflects the significant diversity in network patterns among PWA, which also underscores the importance of individualized brain stimulation in this population.

In the second phase, a single-session double-blinded sham-controlled trial was conducted to the same group of PWA to examine the immediate neuromodulatory effects of the individualized dual-site tACS with concurrent SLT. In summary, we found that sham condition did not result in significant improvements in any neurological or language outcome measures, indicating that SLT combined with the placebo effect alone was insufficient to induce meaningful changes in brain networks or language performance after a 30-min dosage. Among all conditions, only RI stimulation produced substantial positive post-treatment changes across a range of neurological and language outcome measures including target node strength, global efficiency, modularity, small-worldness, and divergent naming performance. RI stimulation also induced larger increase in modularity than sham stimulation and larger improvement in divergent naming than EA stimulation. Although the small sample size and single-session dosage may substantially reduce the detectability of treatment effect, the observed trend indicates that dual-site high-definition in-phase tACS using this novel targeting approach can induce positive language facilitation effect alongside with global network reorganization.

### 4.1 Language facilitation involves network reorganization rather than dysfunctome restoration

First and the foremost, the current findings suggest that the primary neural engagement effect of dual-site HD tACS is increasing the centrality of the target sites. Yet, directing neural resources to different target sites selected by restoration principle and enhancement principle shows differential network modulatory effects, in which restoration-based targeting has superior effect over enhancement-based targeting. The current target selection methods select target edge with high betweenness, which typically represents a connection with a high likelihood of linking two connector hubs in the network (Sporns et al., 2007). In RI stimulation, a centralized-but-weakly-connected edge within an individual’s dysfunctome was selected, which likely represents two weakened connector hubs were selected. Since small-world architecture depends on effective integration between connector hubs from different network modules (Fornito et al., 2016), enhancing the relative centrality of these weakened connector hubs–through certain mechanisms of brain adaptability–appears to facilitate the reorganization of the overall functional network architecture. This reorganization results in a more integrated, specialized, and small-world-like network (i.e., a highly clustered yet integrated architecture), which may facilitate language processing. However, it is important to note that RI stimulation did not increase the connectivity of the target edge nor the average connectivity within the target dysfunctome. Together with the findings from correlational analysis showing a trend of negative association between the target dysfunctome and divergent naming performance as well as modularity, we hypothesize that RI stimulation may facilitate language by “redistributing” neural connections from the dysfunctome to other regions and facilitating improvement in global network properties. Further investigation is needed to verify this hypothesis. The potential causal link between the increases in global network properties such as global efficiency, modularity and small-worldness in the theta network and the language facilitative effect in PSA has not been previously reported in the literature. Nevertheless, this finding aligns with previous studies showing that people with aphasia exhibit decreased small-worldness in the theta network compared to healthy controls in EEG (Sims et al., 2016; Dattola and La Foresta, 2022; Mehraram et al., 2023). These studies suggest that the ubiquitous small-world network architecture observed in healthy brain networks (Bullmore and Sporns, 2009) may also play a critical role in supporting language processing in PWA. Although not focused on global network properties, previous findings also suggested that theta oscillatory network in general was linked to language processing (Bastiaansen et al., 2005; Murphy, 2016) and aphasia recovery (Nicolo et al., 2015). For example, Nicolo et al. (2015) demonstrated that post-stroke language recovery was correlated with increased node degree in right Broca homologue within the theta EEG network independent of age, initial lesion size and clinical severity. The current findings further reinforce the notion that enhancing the global network properties in theta network may improve language performance in PWA. However, further investigation is needed to verify this hypothesis.

This finding challenges the neurological feedback cycle we hypothesized. In other words, the dysfunctome helps to identify a stimulation target that does not engage the dysfunctome itself. It seems counter-intuitive, but this observation leads to the hypothesis that dysfunctome examination aids in pinpointing key hubs in the network, but recovery is likely based on certain functional reorganization “beyond” the dysfunctome rather than restoration of the dysfunctome itself.

Alternatively, EI stimulation, which targets a centralized-and-strongly-connected edge within an individual’s dysfunctome, likely selects two intact connector hubs. These connector hubs hold the characteristics of being centralized in the aphasia-predictive dysfunctome, while being strongly connected at the same time, which might represent the key regions of the optimal neural pathways within the individual’s adaptive reorganized network after stroke. Therefore, enhancing the centrality of these intact hubs was not necessarily linked to substantial restoration of global network properties, which may undermine the overall language facilitative effect.

The exact mechanisms underlying both stimulation approaches remain unclear. However, both observations align with the hypothesis that language recovery in PSA relies on a complex, distributed network that integrates both the residual canonical language regions and newly-recruited domain-general regions (Sims et al., 2016; Kiran and Thompson, 2019; Kiran et al., 2019). In fact, research indicates that greater tissue damage within canonical language regions reduces the potential for their restoration, thereby increasing dependence on extra-brain regions for recovery (Turkeltaub et al., 2011; Sims et al., 2016). This may explain why disrupted connections within the EEG dysfunctome show limited potential for restoration and are less responsive to stimulation. Therefore, it is hypothesized that rather than attempting to restore the dysfunctome itself, redirecting neural communication to broader, intact brain regions may offer greater potential for facilitating language recovery.

### 4.2 Differential effects of in-phase and anti-phase stimulations

It is also worth noting that the anti-phase stimulation (EA condition) produced small but noticeable increases in the connectivity of the target dysfunctome, whereas such an increase was not observable in other conditions. As discussed earlier, improving the connectivity within the dysfunctome was not associated with language facilitative effect, however, these results indicate that anti-phase stimulation might have more focal modulatory effect to the target network.

Although earlier studies proposed that in-phase stimulation synchronizes oscillatory activities between brain regions and anti-phase stimulation decouples them (Polanía et al., 2012; Helfrich et al., 2014), more recent studies presented conflicting findings that anti-phase stimulation may actually increase synchronization between brain regions (Salamanca-Giron et al., 2020; Zhang et al., 2023), because the anti-phase waveforms may align with the optimal wave propagation lag between distant brain regions (Salamanca-Giron et al., 2020). Zhang et al. (2023) proposed that “zero-lag synchronization” may not always be beneficial to signal processing. This raises an important consideration that dual-site tACS aiming to modulate connectivity should first determine whether the connection of interest involves “zero-lag synchronization” or “phase-lag synchronization.” Selecting the appropriate stimulation approach that aligns with the specific connection type could optimize the target engagement effect. In the current study, phase lag index was used to model the target dysfunctome, which might explain why anti-phase stimulation had an comparative advantage in increasing the connectivity in the target dysfunctome. Another potential explanation lies in the differential directionality of wave propagation induced by in-phase and anti-phase stimulation. A recent study by Alekseichuk et al. (2019) demonstrated that anti-phase tACS generates unidirectional electric fields at any given moment, with the propagation direction alternating over time between stimulation sites. In contrast, in-phase tACS consistently produces bidirectional electric fields at any given moment. Together with the observations in the current study, it is possible that the unidirectional anti-phase stimulation favors the time-lag propagation between the target sites, but this restricted the connectomic modulatory effect within the dysfunctome, which is not language facilitative (moderate evidence supported that RI had greater language facilitative effect than EA). In contrast, the bidirectional in-phase stimulation could not strengthen the “phase-lag synchronization” within the dysfunctome, but it allowed greater connectomic modulatory effects outside the dysfunctome, which in turn facilitated functional reorganization, resulting in language facilitation. Nonetheless, the current study has limited ability to investigate the exact neuromodulatory mechanisms of in-phase and anti-phase stimulation on EEG connectome in group-level analysis due to the individualized nature, thus, this hypothesis needs further verification.

### 4.3 Strengths, limitations, and future of the current individualized targeting methodology

#### 4.3.1 Strengths

The current individualized targeting methodology was based on a combination of group-level dysfunctome identification, individual dysfunctome examination, and edge prioritization by pre-defined target selection principles. This method had proved its ability to select different individualized targets for different individuals in the heterogeneous sample we recruited.

Compared to the existing theory-driven individualized targeting methods, this approach does not rely on pre-defined targets identified by other studies or established theories. Instead, it employs a data-driven strategy, which is especially advantageous for diseases with heterogenous subtypes that are difficult to categorize based on the multifaceted clinical features. Additionally, the state-dependent nature of this method is beneficial for conditions that involve intraindividual changes over time, such as stroke or progressive diseases. Furthermore, this method ranks potential stimulation candidates using a priority index, making it particularly useful when certain stimulation locations are medically contraindicated (e.g., due to craniectomy, surface wounds, or metallic implants). In such cases, the next prioritized target that is suitable for stimulation can be selected.

#### 4.3.2 Limitations

Despite its strengths, this method has several limitations. The small sample size in this study limits the generalizability of the results, especially when the sample was heterogenous. Also, the use of EEG sensor-space network as neural measurement instead of using source-space approaches could undermine the spatial precision of modeling and stimulation. Yet, the main purpose of this study was to demonstrate the feasibility of this novel, low-computation-loading, EEG sensor-space dysfunctome-guided targeting for dual-site tACS. This simple methodology builds on the assumption that EEG sensor-space and tACS can form a causal feedback cycle without understanding the underlying neural sources. According to our current findings, we support the feasibility of this methodology by demonstrating reasonable identification of individualized target, safety and tolerability, successful blinding, preliminary target engagement effect, and preliminary clinical benefit. Another limitation concerns the test-retest reliability of EEG networks. If certain edges exhibit unstable connectivity over time, the selected target may vary significantly across time points. To address this issue, regular reassessments or a closed-loop system for online target selection may be necessary to optimize the state-dependent target selection process.

#### 4.3.3 Future directions

As the first attempt in using EEG dysfunctome to inform tACS target selection, there are several aspects of this methodology that require further exploration. First, the choice of which dysfunctome to target can induce vastly different effects, as the modulatory effects of tACS are considered to be state-dependent (Feurra et al., 2013). In other words, the participant’s engagement in a specific brain state can significantly influence the outcomes of tACS. In the current study, an AQ-predictive dysfunctome was identified during a divergent naming task and used as the target. However, because AQ is a multifaceted clinical measure, it remains unclear whether the selected SLT used in the study could effectively prime a brain state that favors the modulatory action of tACS. To optimize the use of dysfunctome-based tACS, it would be beneficial to identify a target dysfunctome under a focused and reproducible brain state. For example, a “naming-predictive” dysfunctome identified during a naming task could serve as the target. In this scenario, the dysfunctome-based tACS could be paired with SLT specifically designed to focus on naming. This approach would better align the therapy with the target dysfunctome, ensuring optimal effects from the tACS.

Second, the optimal edge prioritization method also remains unclear. For example, using edge betweenness centrality as a priority indicator is just one of many possible approaches for determining the relative importance of an edge within the network (Bröhl and Lehnertz, 2022). The choice of priority indicator can significantly influence the identification of stimulation targets, meaning that the targets could vary substantially if a different indicator were used. This highlights the need for further investigation into the most effective priority indicators for target selection.

Third, this study did not demonstrate that tACS could alter the connectivity of the target edge or dysfunctome following a single session. Instead, substantial changes were observed in the target node strength across all three real stimulation conditions, highlighting that tACS has a more consistent target engagement effect on node strength. From this perspective, it may be worth investigating whether target selection should focus on nodes rather than edges. Nonetheless, the current study only tested single-session dosage, it is also possible that dysfunctome change requires longer-term treatment and higher dosage.

Lastly, as this study shows that tACS can modify global network properties to achieve clinical facilitative effects, it would be valuable to explore network-property-based target selection methods. For example, selecting targets that could possibly maximize a certain symptom-related global network property within the network could be a promising direction for future research.

## 5 Conclusion

This study was the first-of-its-kind, dysfunctome-based, data-driven individualized tACS clinical trial. The findings suggested that combining EEG dysfunctome identification, individual dysfunctome examination, and priority-based targeting principles can effectively guide the selection of individualized stimulation targets for dual-site in-phase tACS. A single-session of individualized in-phase tACS adopting restoration-based targeting principle successfully enhanced the target node strength and global network properties, and facilitated language performance in people with PSA. The language facilitation observed in restoration-based stimulation is potentially attributable to network reorganization following elevation of the functional role of the weakened connector hubs. This study highlights the significant potential of tACS in modulating functional networks and provides a novel methodology for the emerging field of individualized connectomic tACS. Although the small sample size limits external validity, the current feasibility study proves that this approach holds potential for application in longer-term treatment and in various network-based disorders.

## Data Availability

The raw data supporting the conclusions of this article will be made available by the authors, without undue reservation.
